# Phytogenics in swine nutrition and their effects on growth performance, nutrient utilization, gut health, and meat quality: a review

**DOI:** 10.1007/s44154-024-00209-2

**Published:** 2025-02-07

**Authors:** Muniyappan Madesh, Jin Yan, Gao Jinan, Ping Hu, In Ho Kim, Hao-Yu Liu, Wael Ennab, Rajesh Jha, Demin Cai

**Affiliations:** 1 College of Animal Science and Technology, Yangzhou University, Jiangsu, 225012 China; 2 Department of Animal Resource and Science, Dankook University, Cheonan, 330-714 Republic of Korea; 3 Department of Human Nutrition, Food and Animal Sciences, College of Tropical Agriculture and Human Resources, University of Hawaii at Manoa, Honolulu, HI 96822 USA

**Keywords:** Antibiotic growth promoters, Anti-inflammatory, Growth performance, Phytogenics, Swine

## Abstract

Phytogenic feed additives have undergone extensive testing in swine production to determine their effects on gastrointestinal function and health, as well as their implications for pigs' systemic health and welfare, flock production efficiency, food safety, and environmental impact. These feed additives derived from plants, encompassing herbs, spices, fruits, and various plant components, contain numerous bioactive ingredients. An examination of published documents concerning the supplementation of phytogenic feed additives uncovers conflicting findings about their efficacy in swine production. This suggests that additional effort is required to ascertain the suitable inclusion levels and thoroughly clarify their mechanisms of action. This review aims to summarise the prevailing trends in the application of phytogenic feed additives in poultry, emphasising their effects on growth performance, nutrient digestibility, biochemical profiles, gene expression, hypocholesterolemic properties, immunity, meat quality, fatty acid composition, amino acid content, and the gut microbiota of swine.

## Introduction

An excellent first step in addressing the alleged antibiotic resistance issue was the 2006 European Union-wide ban on using antibiotic growth promoters (AGP) in farm animals (Millet and Maertens 2011). Antibiotics have continued to be used as a growth promoter in many nations, even though several jurisdictions followed suit due to the absence of quantitative monitoring data and scant regulation on AGP (Sampath et al. 2023). However, considering that antibiotic resistance is a growing public health concern, it's critical to develop different strategies for raising healthy animals (Jha and Berrocoso 2015). Furthermore, eliminating the use of AGP has resulted in adverse effects on swine health and performance and a rise in the prevalence of animal diseases (Muniyappan et al. 2021; Sampath et al. 2023). After AGP was eliminated from feeding programs, enteric diseases rose to the top of the animal industry's list of worries. The industry has been suffering from inadequate manufacturing efficiency, excessive bacterial development in the small intestines, poor vitamin absorption, and related food contamination (Kikusato 2021; Li et al. 2012a). Various feed additives in swine have been explored as AGP substitutes with differing degrees of effectiveness (Muniyappan et al. 2021). These often-used feed additives belong to the main categories of organic acids, enzymes, organic minerals, bacteriophages, clay, eubiotics, phytogenics, hyperimmune, probiotics, antimicrobial peptides, and prebiotics as possible substitutes if animal feeds no longer contain AGP (Nguyen et al. 2020).

The use of plant extract, herbal extract, essential oil, and oleoresins in swine has improved continuously over the years due to the high demand for antibiotic-free pork and its well-studied benefits. The phytogenics market reached 500.46 million USD in 2024, and this improved approach to increasing phytogenics in livestock diets is growing the worldwide phytogenics market, that is to say, projected to reach 613.94 million USD by 2029, at a mixture yearly growth rate of 4.17% (Kazempoor et al. 2022). Many herbal products are used as growth-promoting feed additives in livestock production because of their medicinal and functional properties and beneficial effects, such as antimicrobial, gut microbiota, antioxidant, anti-inflammatory, and immunomodulatory activities that do not negatively affect growth and feed efficiency (Zhai et al. 2018; Kuralkar and Kuralkar 2021). Natural products are becoming more and more popular as they are considered to have fewer negative side effects than synthetic ones (Guo et al. 2024). The development of plant-based feed additives has emerged as an important research area, bearing great importance for improving animal husbandry practices and the quality of animal products.

Among the alternatives, dietary phytogenics have been broadly applied globally because of their antimicrobial, anti-inflammatory, and antioxidant properties, which can reduce pH in the gastrointestinal tract (GIT) (Mohammadi Gheisar and Kim 2018), play their part against pathogenic bacteria, and eventually improve growth performance, nutrient digestibility, and decreased fecal gas emission in pigs (Yan and Kim 2012). The dietary supplementation of quillaja saponin increased growth performance, nutrient utilization, meat quality, and fecal microbiota in growing pigs (Muniyappan et al. 2022a). Deng et al. (2007) reported that supplementing phytogenics feed additives in diets could increase growth performance and nutrient digestibility and alter gut microbiota in piglets. The dietary supplementation of chestnut wood extract enhanced growth performance, showed antioxidant and anti-inflammatory effects, and altered gut microbiota in piglets (Biagi et al. 2010). In other studies, under normal physiological conditions, dietary supplementation of phytogenic was favorable to swine to improve performance and control diarrhea in weaning pigs challenged with *E.coli K88* (Li et al. 2015), increased lymphocyte concentrations, and decreased urea nitrogen, diamine oxidase, and lipopolysaccharide in the blood in pigs (Cairo et al. 2018). The process of phytogenics’s action in the animal’s diet is complex. *Quillaja Saponaria* contains triterpenoid (Francis et al. 2002), *Achyranthes japonica* contains phytoecdysteroid, saponin, polysaccharide, inokosterone, and 20-hydroxyecdysone (Muniyappan et al. 2022b), capsicum contains vanillamide alkaloids (Long et al. 2021a), *Forsythia suspense* contains *forsythiaside A*, *forythialan A, Phillyrin* and *Phillygenin* (Long and Piao 2021). Herbal and plant extracts' bioactive components can improve pig digestibility through a variety of processes, including increased digestive enzyme activity (Mohammadi Gheisar and Kim 2018), modulation of the microbiome in the GIT (Peng et al. 2021), and improved gastrointestinal function and health (Szabó et al. 2023). Thus, phytogenics has been proven to increase nutrient utilization while reducing the quantity of undigested material accessible to fermentation in feces of pigs. Ammonia, methyl mercaptans, hydrogen sulfide, carbon dioxide, and emissions hurt livestock production and agricultural workers; thus, employing plant products to suppress ammonia production is critical for maintaining and improving livestock performance and animal and human health.

Phytogenics are a variety of compounds with a range of biological activities that are thought to provide some benefit comparable to antibiotic growth promoters. Phytogenic feed additives contain pharmacological compounds derived from plants that benefit livestock and animal production. These benefits include improved growth performance, health, reproduction, better product quality, and reduced gas emissions and toxicity. Herbal products are readily available, simple to prepare, and cost-effective. Also, they produce less residue, exhibit less toxicity, and result in fewer side effects in animal product production, making them generally safer for human consumption. However, the efficacy and processes of phytogenic feed additives have not been fully investigated because plants contain varying amounts of pharmacologically active chemicals that may vary from batch to batch. Known mechanisms of action include antimicrobial, immunomodulatory, antioxidant, and gut microbiota-regulatory activities. Combining metagenomics, transcriptomics, proteomics, and network pharmacology tools allows for the elucidation of the properties and functions of phytogenic feed additives and the developing of practical, affordable ways to use plant-derived additives as effective alternatives to antibiotics in animal production.

**Fig. 1:**
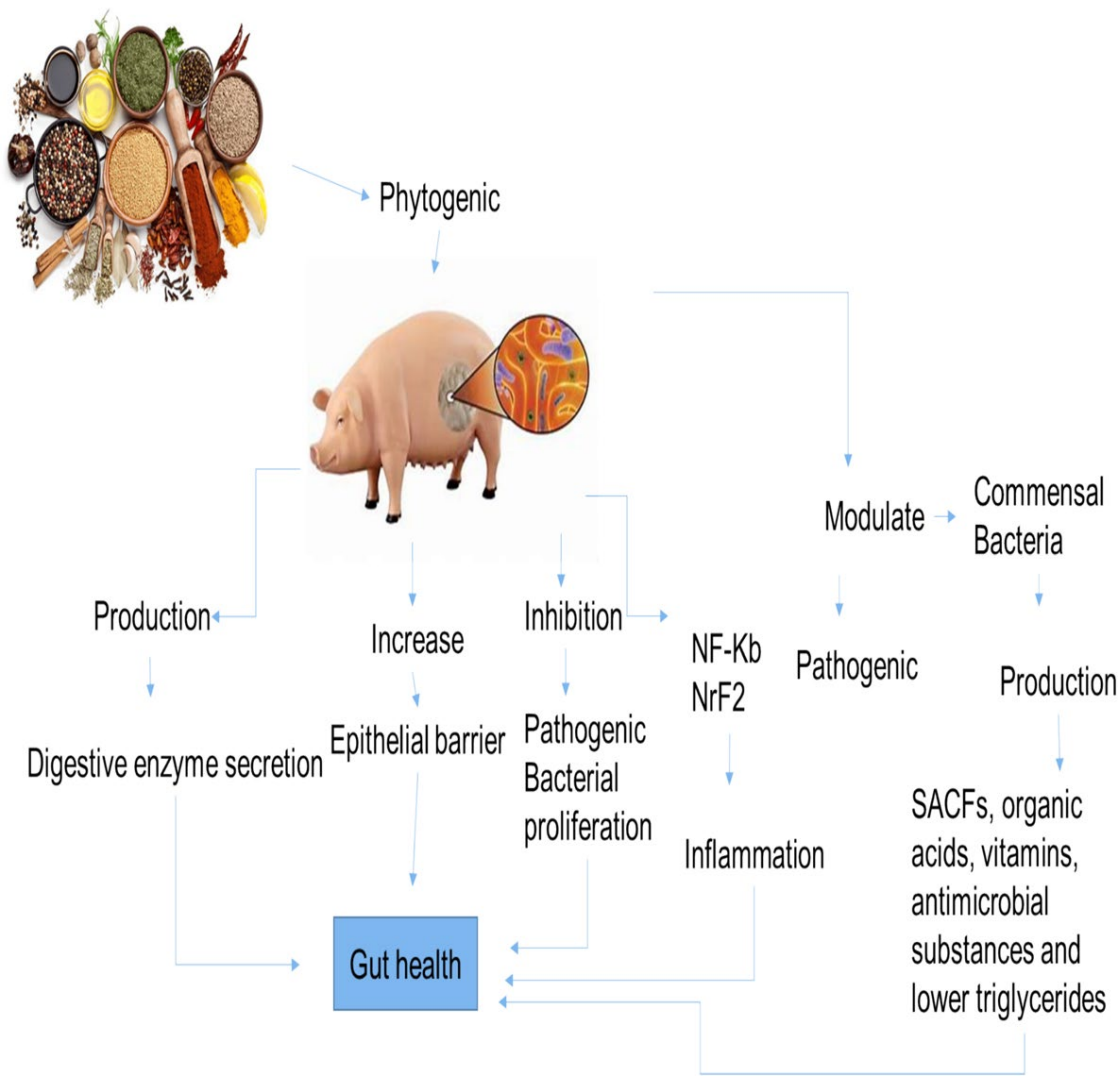
Effect of phytogenics on swine intestinal health

Therefore, it is crucial to review the impacts of phytogenics on nutrient utilization, gut microflora and immunity, and swine's overall health and performance. Overall, the effects of phytogenics on pig intestinal health are presented in Fig. 1.

### Phytogenic feed additive

Plant-derived natural compounds, herbal formulations, plant extracts or biochemical compounds are commonly referred to as phytogenic feed additives (also known as phytobiotics or phytochemicals) to improve feed properties, improve animal production performance and improve the quality of animal products (Wang et al. 2024). Chinese herbal and plant extract feed additives are two terms often used to classify plant components according to their source and processing.

Because of their positive impact on human health, traditional herbal remedies have been used for over 3,000 years (van Wyk and Prinsloo 2020). Livestock animals have been used for over 2000 years (Gong et al. 2014). Producers have gradually recognised the positive impacts of phytogenic feed additives on animal performance, such as improvements in body weight, feed conversion ratio, and meat quality. Later, studies revealed the antimicrobial, antiviral, antifungal, antioxidant, and anti-inflammatory properties of the herbs, which played a role in their beneficial effects. As a result, herbs have historically served as complementary or alternative antibiotics intended to promote or treat health diseases. Recently, the diverse roles of these agents in nutrient metabolism, immune responses, and gut health in livestock animals have come to light, improving our understanding and application. Advances in extraction techniques and the identity of active ingredients have increased efforts to explore phytogenic extracts and compounds as alternatives to antibiotics in animal feeds. Herbs used in animal production are generally safe, cost-effective, and environmentally friendly, suggesting their wide applicability. Currently, most animal feed manufacturers incorporate phytogenic feed additives into their pig feed formulations.

### Growth performance

Phytogenics have been reviewed for their ability to grow in commercial swine production since the gradual reduction of AGP in swine diets. AGP prevents intestinal inflammatory cells from producing and excreting catabolic mediators, which lowers intestinal microbiota (Windisch et al. 2008). By contrast, phytogenics growth promoters by altering the intestinal environment and improving the intestinal barrier function through the fortification of favorable gut microbiota, the pathogen’s competitive exclusion, and the immune system activation. During the addition of phytogenics, plant extract, herbal extract, essential oil, and oleoresins from the supplements compete with the pathogenic bacteria in the gut for nutrition; they colonize the intestine, creating an environment in which dangerous bacteria cannot survive, and they release digestive enzymes (such as galactosidase, amylase, etc.), which aid in the enhanced nutritional absorption and improve animal growth performance (Ali et al. 2021). As such, phytogenics way of action differs from antibiotics in animals. Therefore, together, they can increase growth performance. An increase in average daily gain (ADG), average daily feed intake (ADFI), and gain-to-feed ratio (G:F) have been associated with improvements in body weight growth (BWG). Growth performance (ADG and G:F) was better in pigs fed with herbal extracts (Green tea leaves and pomegranate fruit) compared to the control diet (Bontempo et al. 2014). Studies have demonstrated enhanced growth performance in pigs fed a diet supplemented with herbal extract compared to a control diet without such supplementation (Liu et al. 2008; Lan et al. 2017). Adding various phytogenics can have varying effects and performance. Comparative research found that *Scutellaria baicalensis* and *Lonicera japonica*, both as phytogenics, increased ADFI and BWG of pigs. Reyes-Camacho et al. 2020) reported that supplementation with a mixture of phytogenics (eucalyptol, p-cymene, linalool, anethole, and thymol added as essential oils from the Fabaceae, Laminaceae, Schisandraceae, and Zingiberaceae) increased piglets born alive and BW of newly-born piglets. Dietary inclusion with a mixture of herbal extracts (buckwheat, thyme, curcuma, black pepper, and ginger) improved BW, ADG, and G:F in weaning piglets (Yan and Kim 2012). Kong et al. 2007) found increased ADG and ADFI of pigs fed herbal extracts (*Astragalus membranaceus*, *Acanthopanax senticosus*, *Codonopsis pilosula*, *Crataegus pinnatifida*, and *Salvia miltiorrhiza*). The research reviewed the inclusion of some herbal and plant extracts as phytogenics on the growth performance of swine: bitter citrus extract, thymol, carvacrol, sage, nettle, lemon balm, coneflower, *Coptis chinensis*, *Silybum marianum*, black pepper, chestnut wood, *Eucommia ulmoides*, Turmeric, *Yucca schidigera*, and garlic. Several other studies found that a mixture of plant and herbal extracts can be used as phytogenics in commercial swine production as growth promoters (Biagi et al. 2010; Sampath et al. 2020; Yang et al. 2022b; Hsu et al. 2022; Dang et al. 2022). It is reported that the dietary inclusion of green tea leaves and pomegranate fruit improved ADG and G:F compared to basal diets. The dietary supplementation of *Micelle silymarin* increased ADG in finishing pigs (Zhang and Kim 2022). Moreover, it has been demonstrated that essential oil and organic acid combined improve hepatic energy retention and nutrient absorption (Ma et al. 2022a). The dietary supplementation of Yucca schidigera and Quillaja saponaria consumed 5.1 and 6.1% more feed and showed a 7.6 and 8.6% higher ADG compared to control, respectively. The same study resulted in 4.5 and 5.2% higher ADFI and 6.2 and 6.1% higher ADG for Yucca schidigera and mixed Quillaja saponaria and essential oil compared to the control, respectively (Bartoš et al. 2016). Increased nutrient digestibility and growth performance may be the reason for the energy changes (Ma et al. 2022b).

The impact of phytogenic feed additives on the feed intake of pigs remains unclear. The alteration in feed intake compared to the control group as a result of dietary supplementation with phytogenic feed additives varied from − 9% to 12%, according to the review by (Wang et al. 2024). Additionally, a more recent review by (Abdelli et al. 2021) indicated a range of − 3% to 19%. The numerical changes observed from growth performance trials may not serve as conclusive evidence of the animal's preference or aversion to feed supplemented with phytogenic feed additives. This is due to the underlying assumption that increased feed consumption indicates desirability. The identified improvement in feed intake may be linked only to the increased growth rate of the animals, which is often observed with various growth-promoting additives (Windisch et al. 2008). A study by Frankič et al. (2010) indicated no influence of phytogenics inclusion on pig’s growth performance. When piglets were used to investigate the effects of a mixture of essential oils of cinnamaldehyde, thymol, and carvacrol on the gut microbial profile and growth performance, phytogenics inclusion had no impact on BW, ADFI, and FCR (Li et al. 2012a; Hsu et al. 2022). Wang et al. (2020) also reported that there was no significant effect of herbal extract mixture (golden-and-silver honeysuckle, huangqi, duzhong leaves, dangshen) in the feed on the growth performance of piglets. However, dietary supplementation of *Moringa oleifera* and mulberry leaf extracts did not affect growth performance in finishing pigs (Chen et al. 2021). Also, it was noted that the weaned pigs fed diets containing a combination of herbal extracts (buckwheat, thyme, curcuma, black pepper, and ginger) did not show a significant decrease in growth performance (Yan et al. 2012). One can hypothesize that the performance and effects of phytogenic feed additives may be significantly amplified in commercial settings that exhibit lower levels of hygiene than in controlled experimental environments.

### Nutrient digestibility

Phytogenic feed additives use energy and nutrients to develop and proliferate inside their host. Yan et al. (2012) reported that the effects of supplementation with herbal extracts (buckwheat, thyme, curcuma, black pepper, and ginger) increase the digestibility of dry matter (DM) and crude protein (CP) in pigs. Dietary supplementation of *Achyranthes japonica* extract increased the digestibility of DM, CP, and gross energy (GE) in growing and finishing pigs (Liu and Kim 2021, 2023; Kumar et al. 2021). Hossain et al. (2023) reported an increased digestibility of DM, CP, and GE pigs fed on a diet supplemented by *Silybum marianum* seed extract. Dietary *Forsythia suspensa* extract inclusion increased the digestibility of neutral detergent fiber in sows (Long et al. 2021b), the digestibility of ether extracts (EE) and organic matter (OM) in weanling piglets, the digestibility of DM, CP, and GE in sows in late gestation (Long and Piao 2021), and the digestibility of DM, CP, GE, and EE in weaning piglets (Long et al. 2019). Sampath et al. (2020) reported an increased DM digestibility in pigs fed on a diet of black pepper. However, Lan et al. (2017) demonstrated that the adding blend of herbal extract *Astragalus membranaceus*, *Codonopsis pilosula*, and allicin had increased DM and GE in the growing phase, DM, CP, and GE in the finishing phase of pigs. Compared to controls, phytogenics generally enhanced nutrient digestibility across different stages in swine. This increased digestibility of DM, CP, GE, and OM was also reported in a review by Long et al. (2021b). This study examined the impact of including *Forsythia suspensa* and natural capsicum as phytogenics on weaned pig diets. The findings demonstrated that phytogenics enhanced the function of the intestinal barrier and the ratio of villus height to crypt depth, which boosted intestinal absorption and improved nutritional digestibility. Liu et al. (2016) reported an increased digestibility of DM, CP and GE in pigs fed dietary of *Scutellaria baicalensis* and *Lonicera japonica* inclusion.The dietary supplementation of Cinnamaldehyde and thymol significantly improved the nutrient digestibility of dry matter, crude protein, and energy in piglets (Tian and Piao 2019; Li et al. 2012a). The administration of a cocktail of essential oils at a dosage of 250 mg/kg resulted in a notable enhancement in the nutrient digestibility of CP and GE in grower-finisher pigs (Yan et al. 2010b). The ileal digestibility of crude protein and most amino acids showed improvement when an essential oils cocktail (300 mg/kg) containing menthol as a major ingredient was used, whereas a cocktail containing cinnamaldehyde did not provide similar improvements (Maenner et al. 2011). The supplementation of medical plants (Huang et al. 2012; Zhao et al. 2016), *Silybum marianum* (Hossain et al. 2023), and *Quillaja saponin* (Muniyappan et al. 2022a) increased the nutrient digestibility caused by the use of phytogenics. On the other hand, several studies indicate that phytogenic types (ginger, buckwheat, thyme, curcuma, and black pepper) have no impact at all or could lead to harm (Yousfi et al. 2021). Hossain et al. (2024) found that *Achyranthes japonica* extract in the diet did not affect nutrient digestibility in growing pigs. The supplementation of quillaja saponin does not affect nutrient digestibility in growing pigs (Dang and Kim 2021). Dang et al. (2022) also reported that the pigs fed inclusion of *Silybum marianum* did not affect the digestibility of CP, GE, and DM. The inconsistent results might be partly due to differences in the animal age and state, also as the types of phytogenic feed additives used.

### Diarrhea

Pathogens induce diarrhea, which is a frequent ailment, especially in piglets. Muniyappan et al. (2023) found that it affects the piglet’s health, particularly during the weaning stage. Stressful periods like weaning can cause digestive disorders. Post-weaning diarrhea caused by *E. coli* mostly affects piglets at two weeks of age. It is characterized by diarrhea, dehydration, and reduced growth in dead and surviving piglets, resulting in significant economic losses (Muniyappan et al. 2023; Law et al. 2024). Their involvement in regulating immune responses and addressing diarrhoea in pigs is notably important, particularly for weaning piglets, who often experience diarrhoea as a result of stress, dietary adjustments, and the presence of pathogens. Numerous phytogenic feed additives, including curcumin and flavonoids, have been shown to alleviate gut inflammation modulation of pro-inflammatory cytokines such as TNF-α and IL-6. Phytogenic feed additives enhance the integrity of the intestinal barrier promotion of tight junction proteins, a reduction in permeability, and the prevention of bacterial translocation. Some phytogenics promote the production of immunoglobulins (such as IgA) and boost the activity of macrophages, dendritic cells, and T-cells, aiding the fight against infections and supporting gut homeostasis. Phytogenic feed additives such as essential oils (e.g., thymol, carvacrol), tannins, and saponins, demonstrate the ability to inhibit the growth of pathogenic bacteria like *E. coli* and *Clostridium perfringens*, which are prevalent causes of diarrhoea. The dietary supplementation of quillaja saponin reduced diarrhea scores in pigs (Muniyappan et al. 2022a). However, the adding essential oils significantly decreased diarrhea scores in weaning pigs (Ma et al. 2022a). Several studies reported that phytogenics could alleviate the harmful effects of *E. coli*-induced diarrhea in weaned piglets (Deng et al. 2007; Gessner et al. 2013). Cytokines are a class of low-molecular-weight proteins produced by mononuclear phagocytes that make up the immune system, help in signal transmission between cells, and regulate immune system responses (TARANU et al. 2010; Guan et al. 2019). Pro-inflammatory cytokines such as tumor necrosis factor α (TNF-α) and interleukin 6 (IL-6) are associated with tissue damage (Ding et al. 2021). Evidence suggests that the reduced pro-inflammatory cytokines (IL-1, IL-6, and TNF-a) promotes the preventive effect of Jiawei Xianglian Decoction on irinotecan-induced diarrhea (Lu et al. 2020). The results showed that in the *E. coli*-induced diarrhea piglet model, stevia residue extract significantly inhibited *E. coli*-induced improvement in intestinal permeability, increased superoxide dismutase (SOD), total antioxidant capacity (T-AOC), glutathione peroxidase (GSH-PX), and reduced malondialdehyde (MDA), and lowered *E. coli* counts in gut microbiota (Liu et al. 2022b). Dietary *forsythia suspense* extract reduced diarrhea and the duration of diarrhea, increased serum IgA and IgG levels, and inhibited *E. coli* colonization in the gut (Long et al. 2019). Zhao et al. (2016) reported a decrease in diarrhea in piglets fed herbal extract supplementation, increased *Lactobacillus* count, and decreased *E. coli* in the fecal microbiota. Wei et al. (2017) reported that in the *E. coli*-induced diarrhea piglet model, essential oil significantly inhibited *E. coli*-induced reducing TNF-α, IL-6, and IL-1 expression levels and prevented improvement in tight junction protein expression. Dietary inclusion of mulberry leaf extract can lower diarrhea rate, increase activities of glutathione peroxidase and superoxide dismutase and capacity of amylase and lipase, as well as mRNA expression related to intestinal barrier function of mucin-2, occludin, and claudin-1 and reduces potential pathogenic bacteria of the hindgut in piglets (Ma et al. 2023). Xu et al. (2020) showed that dietary essential oils and organic acids combination supplementation in weaning piglets lower diarrhea scores, and decrease the expression levels of TNF-α, IL-6, and IL-10, while intestinal barrier function of occludin and ZO-1, and increased *Prevotella* and *Lactobacillus* and lower *Escherichia* and *Shigella* compared with control diet when challenged with enterotoxigenic *E. coli* F4 (K88 +). In addition, the study demonstrated that lactoferricin B affected the mucosal transcriptome profile, reduced *E. coli* F4AC infection, and reduced *E. coli* infection-related diarrhea in weaned pigs (Hou et al. 2011).

### Gut microbiota

**Fig. 2:**
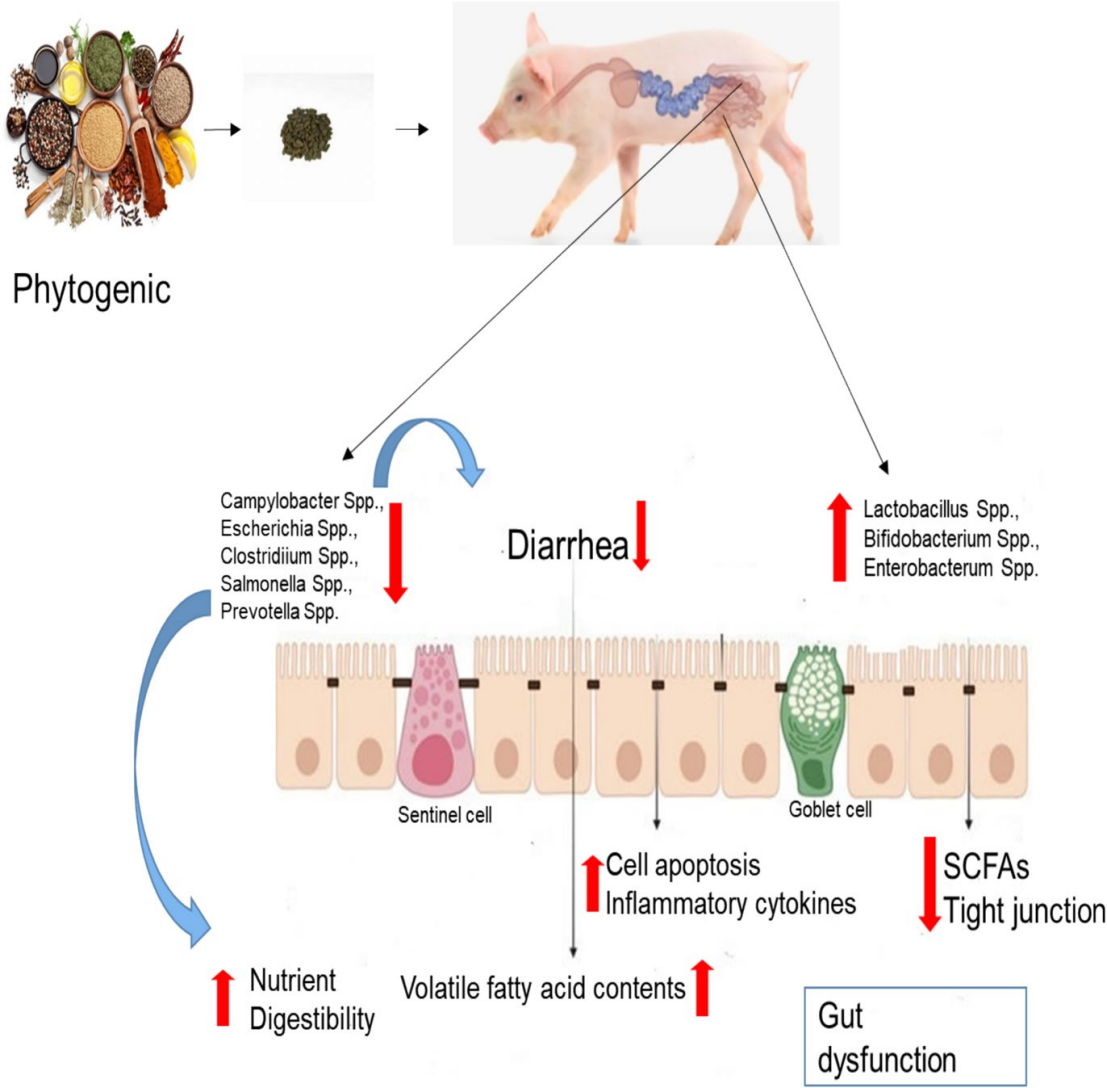
Effect of phytogenics on swine gut microbiota

Despite substantial research, the intricate function that gut microbial communities contribute to the nutrition and development of the host animal remains unclear. In vitro research has demonstrated the well-established antibacterial, antifungal, and antiviral properties of phytogenics (Urbańczyk et al. 2002). It is widely acknowledged that phytogenics have a slightly greater anti-gram-positive than anti-gram-negative bacterial activity (Windisch et al. 2008; Burt 2004). The phytogenics demonstrated dose-dependent effects when propidium iodide was used to test cell integrity in Gram-positive bacteria. On the other hand, most growth inhibition of Gram-negative bacteria did not result in the loss of cell integrity (Thapa et al. 2012). Because phytogenics regulate the gut microbiota in healthy individuals by decreasing the growth of pathogenic species and enhancing beneficial bacteria, they can ameliorate dysbiosis and impact the growth, health, and illness risk of their hosts. The most frequently utilized phytogenics are tannins, flavonoids, saponins, and essential oils (Zeng et al. 2015) and, by competitively excluding harmful bacteria and modifying the immune system in the gut, confer favorable health advantages on the host (Tiihonen et al. 2010). Numerous investigations have shown the impacts of feeding with phytogenics on the microbial fermentation, enzyme activity, and gut microbiota of pigs (Cairo et al. 2018; Wang et al. 2020; Long et al. 2021a). The supplementation of essential oils of thymol and cinnamaldehyde decreased *E.coli* in the cecum, colon, and rectum, lowered total aerobe in the rectum, and improved *Lactobacilli/E. coli* ratio in weaning piglets (Li et al. 2012a), and reduced *E. coli* counts and diarrhea scores (Li et al. 2012b). Cairo et al. (2018) reported that piglets fed the diets with red pepper essential oil increased *Lactobacillus* counts, reduced *Enterobacteria* counts and incidence of diarrhea. Silva Júnior et al. (2020) reported a decrease in *Escherichia-Shigella* and *Campylobacter* in pigs fed dietary inclusion essential oils of eugenol, thymol, and piperine. The supplementation of herbal extracts significantly decreased *E.coli* in pigs (Bontempo et al. 2014). Kafantaris et al. (2018) reported an increased lactic acid bacterium and *Bifidobacteria* and decreased *Enterobacteriacae* and *Campylobacter jejuni* in the feces of pigs fed dietary herbal extracts supplementation. Fecal *Lactobacillus* counts increased and *E. coli* counts decreased in the feces of pigs fed diets supplemented with *quillaja saponin* (Muniyappan et al. 2022a), *Achyranthes japonica* extract (Liu and Kim 2021; Kumar et al. 2021), and *Silybum marianum* (Hossain et al. 2023). Lan et al. (2017) reported that the dietary herbal extract in the pig’s diets improves *Lactobacillus* and lowers *E. coli* and *Salmonella*, possibly due to increased nutrient digestibility. A higher number of *Enterobacteriaceae*, *Lactobacillus spp*., *Bifidobacterium spp*., and lower number *Clostridium spp*., and *Salmonella spp*. and increased isobutyric, propionic, isovaleric acid and total branched fatty acid were found in pigs fed with Alliaceae extract (Sánchez et al. 2020). The evidence demonstrated that stevia residue extract in piglets increases the relative abundance of *Lachnospiraceae*, *Prevotellacea*, and *Coriobacteriaceain* family, and *Prevotella* and *Roseburia* genus and isovaleric acid and isobutyric acid contents of the colon (Liu et al. 2022b). Su et al. (2018) reported an increase of *Bifidobacterium*, *Lactobacillus,* and total bacteria count, a decrease of *Escherichia coli*, and an improved concentration of propionic acid in the colon and cecum, which helped to reduce total serum cholesterol levels and improve the weight gain rate due to addition of a plant essential oil. Dietary supplementation of mixed essential oil and organic acid increased the abundance of *Faecalibacterium* and *Muribaculaceae* in the cecum and *Streptococcus* and *Weissella* in the colon and modulating the cecal and colonic microbial community in pigs, thus increasing animal health (Ma et al. 2022a). Xu et al. (2020) reported that mixed essential oil and organic acid inclusion in the piglet’s diets increased *Lactobacillus* in ileum, cecum, and colon compared with control when challenged with *enterotoxigenic Escherichia coli F4 (K88* +*)*. According to Ma et al. (2022b), adding mulberry leaf powder resulted in a decrease in *Campylobacter* and an increase in *Bifidobacterium* and *Lactobacillus* in feces. Liu et al. (2022a) reported that dietary inclusion of *Forsythia suspensa* could improve the abundance of *Bifidobacteriales* and *Desulfovibrionales* (order level), *Lactobacillaceae, Desulfovibrionaceae Moraxellaceae, Bifidobacteriaceae* and *unclassified_o__Betaproteobacteriales* (family levels) and *Lactobacillus, Acinetobacter, Bifidobacterium, Desulfovibrio, Ruminiclostridium_1,* and *unclassified_o__Betaproteobacteriales* (genus levels) in fihishing pigs (Liu et al. 2022a), increased abundance of *Lactobacillus, Intestinimonas, Rikenellaceae_RC9_gut_group, Roseburia, Butyrivibrio, Mogibacterium* and *Ruminococcus_torques_group* in sows (Long et al. 2021b), increased abundance of *Firmicutes* (phylum level), *Lactobacillus* (genus level) and decreased abundance of *Prevotellaceae_NK3B31_group* (genus level) in cecum and colon as well as improved growth performance of nursery pigs (Long and Piao 2021). The dietary inclusion of natural capsicum extract could increase abundance of *Faecalibacterium, Lachnospira, Eubacterium_ruminantium_group,* and *unclassified_f_Veillonellaceae* and lower abundance of P*eptococcus* in genus levels (Long et al. 2021a). Eventually, this increased the propionic acid, butyric acid and total VFA contents in colon and cecum and decreased acetic acid in cecum with rise in intestinal mucus secretion, it boosts defenses against infections. A study by Biagi et al. (2010) found that piglets given an herbal extract made of Chestnut wood promoted higher relative abundance of *Lactobacillus* in jejunum compared to the control group. These results are particularly of interest because *Lactobacillus* are approved to have beneficial effect on the gut by regulating the composition of gut microflora, developing gut immunity and promoting overall intestinal health (Dávila-Ramírez et al. 2020). Probiotic-induced alterations in the microbial populations of the GIT result in enhanced synthesis of short-chain fatty acids (SCFA) and immunomodulation, both of which strengthen energy metabolism (Nguyen et al. 2018). In short, phytogenic compounds have shown considerable inhibitory properties on harmful microbes in the intestine. Phytogenic compounds such as essential oil, plant extract and herbal extract exhibit incredible broad-spectrum antibacterial effects against gut microbiota. Phytogenic ingredients have the potential to substitute antibiotics in the feed business by modulating gut microbiota and inhibiting colonization by pathogenic bacteria, thereby enhancing animal health. These results provide support to the concept that phytogenics influence the gut microbiota (Fig. 2).

### Immunity

Pathogens must overcome several challenges to colonize the intestinal tract and result in an infection. Physical limitations, including a low pH in the stomach and a short small intestine transit time, are crucial. To effectively initiate an infection, pathogens also need to cross the physical barrier of the epithelium, the gut microbiota inhibitory effects, and the host immune system's reaction. Recent publications demonstrate that certain species of non-pathogenic intestinal microbiota communicate with the epithelium and immune system, modulating the tissue physiology and the ability to respond to infection. One important consequence of probiotics is the modification of the intestinal microbiome, which is also thought to be the foundation for other probiotic advantages. In the gut-associated lymph node, intestinal epithelium cells and dendritic cells serve as mucosal sentinel cells. Phytogenic bind to Toll-like receptors (TLR) in sentinel cells, activating the Nuclear factor kappa-light-chain-enhancer of activated B cells (NF-κB) and mitogen-activated protein kinase (MAPK) pathways (Zhou et al. 2014). The Akt and MAPK pathways regulate intestinal innate immunity by phosphorylating inhibitory κB kinases, activating the NF-κB pathway (Perkins 2007). Gut microbiota trigger the NF-κB pathway, leading to pro-inflammatory responses in the host (Ren et al. 2013). Activation of these pathways leads to increased production of TNF-α, interleukin 1β (IL-1β), IL-6, monocyte chemotactic protein-1 (MCP-1), and interferon-gamma (INF-γ) (Neurath 2014; Pinto et al. 2009). Phytogenics stimulate immune pathways that enhance cytokine production, contributing to a stronger immune defense (Muniyappan et al. 2023). Many phytogenics and their herbal and plant extracts have been shown to modulate the immune system by increasing the number of T/B lymphocytes, the tumoricidal activity of natural killer cells, their mitogenic activity, the phagocytic capacity of mononuclear cells, the induction of cytokines, and ultimately the host immune response to pathogens (Xue et al. 2020). The synthesis of cytokines by macrophages, such as IL-1β, interleukin 2 (IL-2), IL-6, interleukin 10 (IL-10), interleukin 12 (IL-12), and TNF-α, can be influenced by phytogenics (Abdelli et al. 2021). Through stimulation of the immune system, the presentation of antigens, and the production of antimicrobial agents, this activation results in the overexpression or inhibition of genes that control the inflammatory response in addition to cytoprotective effects (Pandey et al. 2023). A higher epithelial barrier, a greater adherence of beneficial microbes to the gut mucosa, and the simultaneous reduction of pathogen adhesion are further advantages (Mohammadi Gheisar and Kim 2018). A study by Li et al. (2012a) reported that pigs fed supplemented by essential oil (thymol and cinnamaldehyde) showed downregulated serum IL-6 and increased serum Lymphocytes, Insulin-like growth factor 1 (IGF-1), and TNF- α compared to the control. Meanwhile, IL-6 may activate the conventional complement pathway. It increases the quantity of complement 5a and the permeability of blood vessels, which causes tissue injury. TNF is categorized as TNF-α, TNF-β, and TNF-γ. TNF-α can trigger inflammatory responses (Zhang et al. 2018). Mononuclear cells such as lymphocytes, macrophages, and the inoblast are all recognized to secrete it (Chen et al. 2023). Reyes-camacho et al. (2020) demonstrated that dietary inclusion of phytogenic actives showed increase an overexpression of barrier function MUC2, immune response peroxisome proliferative activated receptor gamma (PPARγ), coactivator 1 alpha, TNF-α, IL-10, TGF-β1 genes and digestive enzyme Indoleamine 2, 3- dioxygenase. Long et al. (2021a) reported that the natural capsicum extract could increase IL-10 and decrease IFN-γ, IL-6 and TNF-α in serum. Similarly, a decrease in TNF-α and IL-1β and increased immune response were observed by the addition of natural capsicum extract (Liu et al. 2012). Mendivil et al. (2019) found that natural capsicum extract reduces TNF-α, IL-1β, and IL-6 gene expression, alleviating inflammatory responses. Capsicum extract could reduce IL-10, TNF-α, IL-1β, IL-6, and IL-12 by inhibiting the NF-ĸB pathway (Zhou et al. 2014). Oxidative stress represents a well-documented mechanism underlying inflammation and tissue damage. Nrf2 is an important transcription factor that regulates cellular oxidative responses by regulating gene expression for various phase II detoxification enzymes and antioxidants. In the Nrf2 activation process, reactive oxygen species (ROS) play an important role by modifying Nrf2, resulting in its dissociation and subsequent accumulation. Notably, ROS has been proposed as a significant mechanism that primes or activates the NLRP3 inflammasome, although there is some controversy surrounding this topic (Kasai et al. 2020). We proposed that Nrf2 acts as an effector molecule that participates in the inflammatory process mediated by ROS. When the binding sites of Keap1 are fully occupied by Nrf2, the newly produced Nrf2 translocates into the nucleus and induces the transcriptional activation of target genes that contain antioxidant response elements (AREs) in their regulatory regions (Yamamoto et al. 2018). The dietary inclusion of *micelle silymarin* increased 17β-estradiol and prolactin hormone and reduced serum concentrations of IL-1β and TNF-α mediators NF-κB and Nuclear factor erythropoietin-2-related factor (Nrf2) in duodenal mucosa resulted in lower risk of intestinal diseases (Jiang et al. 2020). Su et al. (2018) reported that the inclusion of essential oil could improve the gut barrier of pigs by inhibiting the TLR4/NFkB signal pathway and activating the Nrf2 signal pathway. Yan et al. (2011a) found that the herbal extract can increase the lymphocyte and modify the microorganisms present in the gastrointestinal tract of pigs. The dietary supplementation of forsythia suspensa extract could decrease the level of inflammatory cytokines TNF-α, and improve protein expression of occludin in jejunal on piglets (Long and Piao 2021); lower level of inflammatory cytokines IL-8, increase contents of gonadotropin-releasing hormone (GnRH), but the increased level of GSH-Px and Immunoglobulin A (IgA) in serum on piglets, increased level of inflammatory cytokines IL-6, increased IL-10, but improve the level of GSH-Px and catalase (CAT) in serum on sows, and the inflammatory response and the level of IL-6, IL-10, TNF-α decreased in colostrum, but the level of T-AOC, SOD, and MDA and Immunoglobulin M (IgM) increased (Long et al. 2021b). Suresh Kumar et al. (2021) reported that the dietary inclusion of Achyranthes japonica extract can increase IGF − 1, growth hormone and protein, positively influence health and growth. The evidence demonstrated that stevia residue extract in weaning piglets improved T-AOC, T-SOD, CAT and GSH-Px and lowered MDA in serum and liver, while intestinal enzyme activity of trypsin and amylase were increased in duodenum (Liu et al. 2022b). Supplementation of grape seed to basal diet decreases transactivation of NF-κB (intercellular adhesion molecule 1, chemokine, ligand 2, TNFα, IL8 and serum amyloid A) and Nrf2 (glutathione peroxidase 1, NAD(P)H dehydrogenase, quinone 1, peroxiredoxin 6, superoxide dismutase 1 and thioredoxin reductase, and a reduced expression of several target genes within these transcription factors (Gessner et al. 2013). Other investigators have studied the amounts of secretory immunoglobulin A (SIgA), IgM, and polymeric immunoglobulin receptors in piglets, which play crucial roles in immune maintenance (Zhao et al. 2022). SIgA protects and regulates intestinal mucosal epithelia by limiting the availability of various bacteria and mucosal antigens, whereas IgG directly contributes to an immune response by neutralizing poisons and viruses. Supplementation with resveratrol increases SIgA and polymeric immunoglobulin receptor levels seen in the jejunum and ileum of piglets (Hong et al. 2024). The study reported that g pigs fed a diet supplemented with tea tree oil improved expression of heart fatty acid-binding protein, growth acceleration hormone, and insulin-like growth factors –I and reduced calpain-1 and myostatin gene.

In piglets under challenging conditions such as enterotoxigenic *E. coli F4 (K88* +*)*, Xu et al. (2020) reported that a mixed organic acid and essential oil reduced serum TNF-α, IL-6 and IL-10 and protein expression of zonula occludens-1 (ZO-1) and occludin in jejunum, but protein expression of ZO-1 and occludin in ileal. Essential oil increased levels of intestinal ZO-1 and occludin proteins in pigs infected with enterotoxigenic *E.coli* F4 (K88), reduced levels of INF-γ, MCP-1, IL-6, IL-1β, and TNF-α in the intestines, inhibited the activation of JNK, ERK1/2, and Akt, and lowered the NF-*κ*B protein of the jejunum (Zou et al. 2016a). The addition of essential oil (a bland of carvacrol and thymol) piglets diet could increase gut health and growth performance, reduce inflammation of TNF-α, and regulate intestinal morphology through the TLR4-mediated NF-κB depending signaling pathway (Wei et al. 2017). Some inflammatory cytokines, like TNF-α and IFN-γ, are associated with improved permeability by inducing the tight junction proteins to be endocytosed (Neurath 2014). It has been found that piglets fed the diet plant essential oil had improved enzyme activity lactase and sucrase and increased expression of occludin, glucose transporter 2, and sodium-glucose cotransporter 1 in the duodenum and jejunum (Su et al. 2020). Wang et al. (2020) reported the increased expression of nutrient transporters SLC15A1, SLC5A1, and SLC6A9 in the ileum due to herbal extract supplementation. In this study, Pigs receive nutrients, such as fatty acids, glucose, and peptides, primarily through the small intestine. Furthermore, the small intestine regulates amino acid transport and meets the amino acid requirements of all tissues. According to Bröer and Fairweather (Bröer and Fairweather 2018), SLC15A1 has a high supply of peptides, resulting in a low demand for amino acids. SLC6A19 is an important neutral amino acid transporter in the intestinal apical membrane (Bröer et al. 2004). SLC1A1 is a cotransporter of Na + and glucose across the intestinal brush border membrane (Wang et al. 2020). Goblet cells in the GIT create mucin, which may enhance the gut barrier by preventing pathogenic bacteria from passing through the dense mucus layer. Phytogenesis has been demonstrated to boost the number of goblet cells, which release both mucin and protein barrier factors, thereby protecting cells in the intestinal epithelium. Similarly, herbal extract administration improved the antioxidant activity and function of intestinal barriers in piglets (Silva Júnior et al. 2020). Huang et al. (2022) found that adding the diet of pigs with a combination of plant essential oils, like eucalyptus oil, oregano oil, thyme oil, lemon oil, garlic oil, and coconut oil increases Glucose Transporter 4, lipoprotein lipase, Carnitine palmitoyltransferase I, cluster of differentiation 36, fatty-acid-binding proteins and low-density lipoprotein receptor in liver, as well as the prevention of NF-κB.

Various in vitro and in vivo investigations have shown that phytogenics have immunomodulatory and health-promoting properties. Phytogenic plants can affect the immune system by increasing lymphocyte proliferation, improving macrophage phagocytosis, and influencing the expression of genes of nitric oxide and cytokines like TNF-α, IL-1β, IL-6, and IL-10. According to the existing literature, phytogenic compounds can be employed as immunomodulatory and anti-inflammatory agents and added to animal diets as supplements for enhancing animal health. In summary, the use of phytogenics as feed additives in pig diets has potent effects on the immune response (Fig. 2).

### Biochemical indicators and oxidant status

Several researchers investigated the effect of phytogenics inclusion on biochemical parameters that contribute to nutrition metabolism and the body’s physiological status (Wang et al. 2024). Dietary inclusion of herbal extract could improve white blood cells, IgG, and IgA in serum in finishing pigs (Lan et al. 2017). Yang et al. (2022a) reported that tea tree oil supplementation increased serum alkaline phosphatase, IgG, and IgM, and reduced serum aspartate transaminase in pigs. Li et al. (2012b) reported an increased lymphocyte, phagocytosis, albumin, total protein, IgG, IgM and IgA, C3 and C4 in serum due to dietary adding essential oil. The dietary supplementation of *forsythia suspensa* extract had increased serum IgA, RBC in weaning pigs (Long and Piao 2021). Feng et al. (2023) found that the grape seed proanthocyanidin extract supplementation in pig’s diets increased T-AOC, GSH, and GSH-Px and lowered MDA in serum and liver and decreased total cholesterol and serum triglyceride levels and higher serum high-density lipoprotein cholesterol levels. The mechanisms that elucidate the hypocholesterolemic effect of PFAs could be linked to the diminished activity of enzymes that play a role in lipid metabolism, such as 3-hydroxy-3-methylglutaryl-CoA reductase (an enzyme related to cholesterol synthesis), cholesterol-7 hydroxylase, fatty acid synthase, and the pentose phosphate pathway (Abdelli et al. 2021).

The supplementation of phytogenics increased CAT, nitric acid (NO), thiobarbituric acid reactive substances (TBARS), T-AOC, GSH-Px, and superoxide dismutase (SOD) during gestation, TBARS and T-AOC during lactation, and CAT and SOD in piglets (Reyes-Camacho et al. 2020). Long et al. (2020), (Reyes-Camacho et al. 2020) reported an increased GSH-px in piglets; CAT and GSH-Px in sows; T-AOC, SOD, malondialdehyde (MDA) in colostrum due to inclusion of a *forsythia suspensa* extract. Similarly, dietary supplementation of *forsythia suspensa* extract could improve serum levels of SOD and T-AOC in suckling piglets; SOD, T-AOC, GSH-Px level of serum in weaning piglets. In another study, Long et al. (Long et al. 2019) reported that the inclusion of *forsythia suspensa* extract decreased MDA and increased T-AOC, SOD, and CAT in weaning pigs. Serum cholesterol and triglyceride were decrease in pigs when herbal extracts (Dang et al. 2023), *quillaja saponin* (Muniyappan et al. 2022a), *micelle silymarin* (Wei et al. 2022; Zhang and Kim 2022) were added. The inclusion of *micelle silymarin* showed positive effects on the antioxidant status of sows (Xu et al. 2022). SOD, CAT, and GSH-Px enzymes play an important function in the body's defense against oxidative stress (Su et al. 2018) and were improved by adding natural capsicum extract (Long et al. 2021a); herbal extract (Long et al. 2020) in pig diets. MDA levels were also reduced in pigs when natural capsicum extract (Long et al. 2021a), herbal extract (Wang et al. 2020), Essential oils (Li et al. 2012a), organic acid, and essential oil combination (Xu et al. 2018) were included in diets. The supplementation of essential oils can increase T-SOD, T-AOC, and GSH-Px and reduce ROS and TBARS in the serum of pigs (Zou et al. 2016b; Cheng et al. 2018).

### Meat quality

Meat is not only a main source of vitamins, amino acids, and minerals but also a good energy source (Cairns 2009). Pork is a great source of bioavailable iron and zinc, as well as selenium, vitamins D, A, and B, and other minerals. It has also been demonstrated that plant sources cannot replace meat's protein or vitamins (A and B12) (Geletu et al. 2021). In each case, meat quality assessment begins with quality control of raw meat (Willson et al. 2020). The meat quality can be visually assessed by measuring organoleptic characteristics such as color, look, flavor, and taste. Most sensory evaluations of meat are still performed by humans (panel test) because no laboratory analysis or mechanical device exists that can simulate all the actions of biting and chewing and measure or duplicate human perceptions (Cai et al. 2023). However, fresh meat quality can be defined instrumentally, including composition, spoilage, nutrients, water-holding capacity, color, tenderness, flavors, contamination, and functionality (Sampath et al. 2023). The meat color lightness value indicates the level of oxidized myoglobin, the redness value indicates the level and existence of deoxygenated myoglobin in the muscle, and the yellowness value indicates the level of myoglobin oxidized to high-iron myoglobin. Inosine monophosphate levels increase, indicating an improvement in muscle flavor. For example, it is reported that the inclusion of phytogenics can improve Longissimus muscle and decrease 2-Thiobarbituric acid reactive substances (Urbańczyk et al. 2002; Yan et al. 2011b). Ma et al. (2022b) reported an increase in carcass weight and redness of pigs fed on diet inclusion of mulberry leaf extract. The dietary inclusion of mulberry leaf extract improved carcass weight and loin eye area and decreased myosin heavy chain (MyHC) I and MyHCIIb gene expression in longissimus dorsi muscle and biceps femoris muscle, increased DM and CP, and amino acid concentration of histidine, tryptophan, serine and tyrosine increased, and lysine and glycine were reduced (Liu et al. 2019). Similarly, increased carcass weight, pH, redness, and yellowness, lower drip loss, and increased gene expression of MyHCI, MyHCIIa, and MyHC IIx, decreased CP content and improved levels of the amino acid (Alanine, Threonine, Isoleucine, Lysine and Proline) and increased fatty acid composition of n-3 polyunsaturated fatty acid, C22:1n9, C18:1n9c, C18:3n3, C16:1, and C20:4n6 was found by the supplementation of mulberry leaf extract (Chen et al. 2021). Likewise, it is reported that the inclusion of mulberry leaf extract in the pig’s diet improved liver cytochrome P1A1 expression and decreased skatole content in backfat (Sun et al. 2024).

The dietary supplementation of *Achyranthes japonica* extract reduced water-holding capacity and drip loss (Liu and Kim 2021), lowered drip loss, and increased backfat thickness and lean meat percentage (Kumar et al. 2021). Lan et al. (2017) demonstrated that adding a blend of herbal extracts, *Astragalus membranaceus*, *Codonopsis pilosula*, and allicin improved meat color and redness value and decreased lightness in finishing pigs. Huang et al. (2022) found that adding the diet of pigs with a combination of plant essential oils, like eucalyptus oil, oregano oil, thyme oil, lemon oil, garlic oil, and coconut oil increased T-SOD and T-AOC and reduced MDA content, and increased gene expression of Glucose Transporter 4 and reactive oxygen species in longissimus dorsi, as well as High intramuscular fat content, also known as 'marbling fat,' which is associated with increased meat flavor (Muniyappan et al. 2021). Zou et al. (2016b) reported reduced TBARS and reactive oxygen species in longissimus dorsi, improved pH, carcass weight, and opto star, and reduced drip loss in meat from pigs fed on a diet supplemented with a mixture of essential oil and quercetin. Also, it provided some benefits in the color, lightness, yellowness, and lean meat percentage when the diet was supplemented with essential oil (Li et al. 2017). Likewise, Chuan-Shang et al. (2017) showed that pigs fed the diet with essential oil inclusion can increase the fatty acid percentage of n-3 polyunsaturated fatty acid and monounsaturated fatty acid in longissimus dorsi muscle and improve gene expression of oxidative stability, CAT, and AOC in longissimus dorsi muscle and meat quality of tenderness, intramuscular fat increased and lower drip loss. The study observed lower muscle pH, shear force, and cooking loss, enhanced intramuscular fat and live weight, increased C18:2n6t and decreased C16:0 and C12:0 concentration of fatty acid in longissimus dorsi muscle (Yang et al. 2022a).

The study of Han et al. (2024) found that the inclusion of *Eucommia ulmoides* leaf extract can decrease lightness, yellowness, and drip loss, increase pH and meat color score in quality of meat, and increase GSH-Px, SOD activity, lower the content of MDA, improve the concentration of C20:1, C18:1 cis-9, C14:0, monounsaturated fatty acids, and reduce C20:4n-6, C18:3n-6, C18:2n-6, C20:3n-6 and C18:0, polyunsaturated fatty acids, the higher composition of inosine monophosphate, sweet amino acids, and total free amino acids content, the lower expression level of fatty acid translocase, adipose triacyl glyceride lipase, CCAA T/enhancer-binding protein α and carnitine palmitoyl transferase 1B in longissimus dorsi muscle, as well as improve the expression level of carnitine palmitoyl transferase 1B, acetyl CoA carboxylase, fatty acid-binding protein 4 and adipose triacyl glyceride lipase in backfat. It also proved to improve the total number of adipocytes, increase the protein expression level of p-AMPK-α and p-ACC and downregulate the mRNA expression levels of adipogenic genes such as FAS, SREBPS1c and ACC of pig-fed dietary *Eucommia ulmoides* leaf extract supplementation. Feng et al. (2023) found that the dietary grape seed proanthocyanidin extract supplementation in pig’s diets increased T-AOC, GSH, and GSH-Px, lowered MDA, and increased the expression of lipolysis and fatty acid oxidation-related genes while decreasing the expression of lipogenesis-related genes and activating the AMPK signal in finishing pigs. The reasonable explanation is that the digested and absorbed phytogenics may serve as an exogenous antioxidant, increasing the activity of T-SOD, T-AOC and GSH-PX, effectively reducing the amount of lipid peroxidation caused by reactive ROS, lowering the concentration of MDA, better maintaining the integrity of the cell membrane, and ultimately improving the color and water-holding capacity of pork.

### Fecal gas emission

**Fig. 3:**
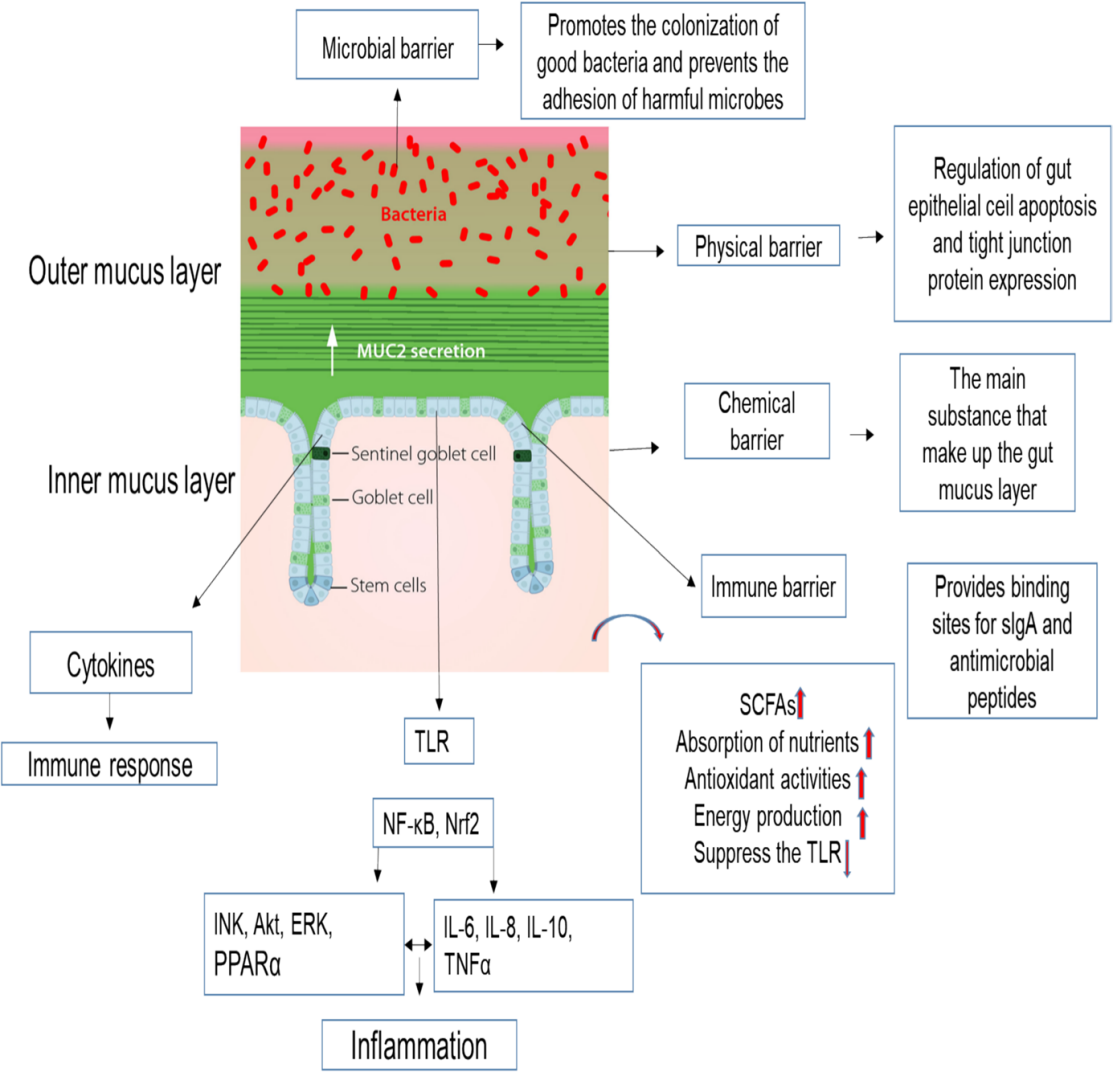
Effect of phytogenics on swine immune response

**Fig. 4:**
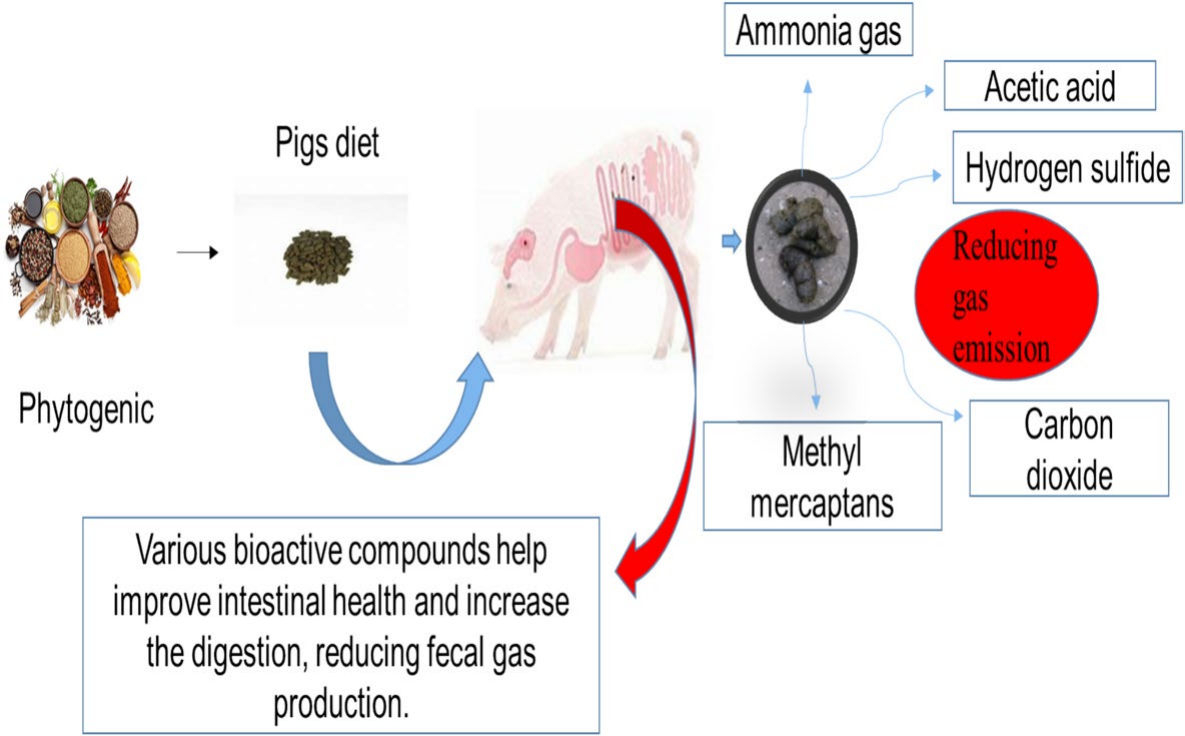
Effect of phytogenics on swine fecal gas emission

The most common noxious gas emissions from livestock farms are methyl mercaptans, ammonia, carbon dioxide, and other odorous compounds chemicals, regarded as severe environmental pollution and pose serious health risks to both animals and workers (Muniyappan et al. 2021). Among these, ammonia emission is intimately related to water and soil acidification, as well as nitrogen deposition in ecosystems. Previous studies suggested that fecal noxious gas emissions could be linked to nutrition retention and the gut microbiome. Muniyappn et al. (2022a) hypothesized that gas emissions from livestock farms are linked to hazardous gut bacteria populations. The microbial breakdown of nutritional molecules in the feces produces ammonia (Li et al. 2012b). When pigs are fed diets supplemented with black pepper, noxious gasses such as ammonia and methyl mercaptans, as well as acetic acid, are significantly reduced (Sampath et al. 2020). Yan and Kim (2012) reported that the inclusion of medical plant extract can reduce coliform bacteria counts and improve *Lactobacillus* counts and nutrient digestibility by decreasing the fecal methyl mercaptans and ammonia. In this study, Muniyappan et al. (2022a) found that dietary inclusion of *quillaja saponin* could reduce fecal gas ammonia and carbon dioxide emissions. The possible reasons for the decrease in ammonia may be attributable to the beneficial effects of the inclusion of herbal extract on the *Lactobacillus* present in the gut (Yin et al. 2018). Suresh Kumar et al. (2021) discovered that adding herbal extracts, particularly *Achyranthes japonica* extract, modulates the microbiota in pig’s gastrointestinal tract. This modulation reduced toxic gas emissions from the pigs. Similarly, Liu et al. (2021) demonstrated that *Achyranthes japonica* extract can increase *Lactobacillus* count and decrease the *E. coli* count in the feces of pigs, hence reducing fecal ammonia and hydrogen sulfide emissions. Yan et al. (2010a) reported that the growing-finishing pigs fed with dietary essential oil lowered fecal gas hydrogen sulfide and ammonia emission. The mitigation of fecal gas emissions from hydrogen sulfide was shown in pigs fed dietary herbal extract supplementation (Yan et al. 2011b). Cho et al. (2005) reported that dietary inclusion of essential oil lower fecal gas hydrogen sulfide emission in pigs. In summary, the use of phytogenics as feed additives in pig diets has potent effects on the fecal gas emission (Figs. 3 and 4).

Phytogenic application along with major physiological responses in swine, are presented in Table 1.

**Table 1:** Phytogenic application with major physiological responses in swine

Feed Additive	Major Components	Species	Beneficial effects	References
Plant extract	Green tea leaves and Pomegranate fruit	Weaning pigs	BW, ADG, and G:F ↑, diarrhea ↓; fecal *Lactobacillus* and *Enterobacteriaceae* ↑, fecal *Escherichia coli* ↓, serum Myeloperoxidase ↑	(Bontempo et al. 2014)
Herbal extract	*Astragalus membranaceus, Codonopsis pilosula* and allicin	Finishing pigs	ADG, G:F, DM and GE ↑, fecal *Lactobacillus* counts ↑, *Escherichia coli* counts ↓, Serum WBC, IgG and IgA concentrations ↑	(Lan et al. 2017)
Herbal extract	buckwheat, thyme, curcuma, black pepper and ginger	Weaning pigs	ADG, G:F, DM and CP ↑, diarrhea ↓, fecal *Escherichia coli* ↓, blood lymphocyte concentration ↑	(Yan et al. 2012)
Herbal extract	*Astragalus membranaceus Bunge, Lycium barbarum L., Atractylodes macrocephala Koidz, Shenqu,* and *Glycyrrhiza uralensis Fiseh*	Finishing pigs	BW, ADG, ADFI and G:F ↑, Serum IGF-I↑, Serum MDA↓, duodenum IGF-IR ↑, liver IGF-IR ↓, meat color, redness and lightness ↓	(Liu et al. 2008)
Phytogenic	Black pepper ( *Piper nigrum*)	Finishing pigs	BW, ADG, G:F, and DM ↑, fecal *Lactobacillus* counts ↑, fecal *Escherichia coli* counts ↓, gas emission of NH_3_, methyl mercaptans, and acetic acid ↓	(Sampath et al. 2020)
Phytogenics	*Fabaceae, Laminaceae, Schisandraceae*, and *Zingiberaceae*	Gestating and lactating sows	Piglets born alive↑, newborn piglets BW↓, colostrum protein and milk fat content↑, milk against *Staphylococcus aureus* and *Bacillus subtilis* ↑, SOD, CAT, GSH-Px↑, IDO↑, MUC2↑, PPARGC1-α, IL-10, TNF-α, and TGF-β1 ↑	(Reyes-Camacho et al. 2020)
Herbal extract	*Lonicera japonica Thunb*, *Astragalus menbranaceus*, *Eucommia folium* and *Codonopsis pilosula*	Weaning piglets	Ileum expression of nutrient transporters of SLC5A1, SLC6A9, and SLC15A1↑, jejunum and duodenum ratio of villus height to crypt depth ↑, duodenum Crypt depth ↓, ileum activities of maltase ↓, ratio of small intestinal weight to BW↓	(Wang et al. 2020)
Essential oils	Thymol and cinnamaldehyde	Weaning pigs	ADG, DM, and CP↑, diarrhea ↓, blood lymphocyte concentration ↑, serum TNF-α, T-AOC, and IGF-1 ↑, serum IL-6↓, colon, rectum, and cecum *E. coli* ↓, colon *Lactobacilli/E.coli* ratio ↑, rectum total aerobe ↓	(Li et al. 2012a)
Herbal extract	*Achyranthes japonica*	Finishing pigs	BW, ADG, DM, CP, and GE ↑, fecal *Lactobacillus* ↑, blood Protein, IGF − 1, Growth Hormone ↑, gas emission of NH3, Total mercaptans ↓, meat drip loss ↓	(Kumar et al. 2021)
Plant extract	*Alliaceae*	growing-finishing pigs	ADG ↑, family *Enterobacteriaceae* ↑, genus *Lactobacillus spp*, *Bifidobacterium spp* ↑, genus *Clostridium spp*., *Salmonella spp*. ↓, Total volatile fatty acids propionic, isobutyric, and isovaleric acids ↑, c2/c3 and (c2 + c4)/c3 ratios ↓	(Sánchez et al. 2020)
Essential oils	thymol and cinnamaldehyde	Weaning male pigs	ADG, DM ↑, serum total cholesterol, and triglyceride ↓, goblet cell ↑, jejunum lactase activities ↑, duodenum sucrose activities ↑, ileum villus and crypt depth ratio ↑, duodenum and ileum expression of occludin and glucose transporter-2 gene ↑	(Su et al. 2020)
Essential oils	thymol and cinnamaldehyde		BW, ADG, and ADFI ↑, G:F ↓, diarrhea ↓, fecal *Lactobacillus* counts↑, fecal *Escherichia coli* ↓, serum Lymphocyte, IgA, IgG, C3, C4 ↑	(Li et al. 2012b)
Essential oils	eugenol, thymol, and piperine	Weaned piglets	ADG, ADFI ↑, nutrient digestibility ↑, fecal score ↓, goblet cells count ↓, colon *Campylobacter* and *Escherichia-Shigella* ↓, colon relative weights and small intestine ↓	(Silva Júnior et al. 2020)
Essential oils	Eugenol and cinnamaldehyde	Growing pigs	Serum Lymphocyte count↑, fecal *Lactobacillus* counts↑, fecal *Escherichia coli* counts ↓, gas emission of NH_3_ and H_2_S concentration↓	(Yan and Kim 2012)
Plant extract	Tea Tree Oil	Finishing pigs	BW and ADG ↑, G:F ↓, serum IgA, IgG, Ig M ↑, serum alkaline phosphatase, GSH-Px ↑, serum aspartate transaminase, MDA ↓, expression of insulin-like growth factors -I, and growth acceleration hormone ↑, and myostatin gene and calpain-1 ↓ in liver and longissimus dorsi muscle, fatty acid concentration of C18:2n6t ↑ and C12:0 and C16:0 ↓	(Yang et al. 2022a)
Herbal extract	*Eucommia ulmoides*	Finishing pigs	Lightness, yellowness and drip loss ↓ and pH and meat color score ↑ (Meat quality), and longissimus dorsi muscle activities in GSH-Px, SOD ↑ and MDA↓, fatty acid concentration of C20:1, C18:1 cis-9, C14:0 ↑ and C20:4n-6, C18:3n-6, C18:2n-6, C20:3n-6 and C18:0↓	(Han et al. 2024)
Plant extract	*Capsicum* extract	Weaning pigs	BW and ADG ↑, diarrhea ↓, digestive enzyme activities in α-amylase, Trypsin, Chymotrypsin ( Ileal and Jejunal mucosa) ↑, serum hormone in β-Endorphin, Growth hormone, 5-hydroxytryptamine ↑, serum in T-SOD, T-AOC, IL-10 ↑, MDA, TNF-α, IL-6, INF-γ ↓, volatile fatty acid contents in Propionic acid, Butyric acid and Total volatile fatty acid (Colon and Cecum)↑, *Faecalibacterium, unclassified_f__Veillonellaceae* (genus) ↑ and *Peptococcus* (genus) ↓	(Long et al. 2021a)
Herbal extract	*Forsythia suspensa*	sows and newborn piglets	ATTD ↑, Litter birth weight ↑, fat, protein contents, T-SOD, T-AOC, IL-10, Ig M, glucose (colostrum) ↑, MDA, TNF-α, IL-6 (colostrum) ↓, Ig A, GSH-Px, Gonadotropin-releasing hormone (piglets serum) ↑, urea, IL-6, IL-8 (piglets serum)↓	(Long et al. 2021b)
Herbal extract	*Forsythia suspensa*	Lactating sows and nursery pigs	ADG, G:F ↑, diarrhea ↓, serum growth hormone, Ig G, SOD and T-AOC ↑ and serum MDA and TNF-α ↓, villus height in the ileum ↑, villus height to crypt depth ratio in the jejunum ↑, expression of occludin (jejunal mucosa) ↑, Lactobacillus in the colon (genus) ↑	(Long and Piao 2021)
Herbal plant	*Macleaya cordata*	Late Gestation	Piglets serum GSH-Px, SOD, CAT, IgG, IgM, and IL-10 ↑ and IL-6, IL-1β, TNF-α and cortisol and MDA ↓, sow serum GSH-Px, SOD, CAT, IgG, IL-10, and IFN-γ ↑ and MDA, IL-1β, TNF-α, and cortisol ↓	(Li et al. 2022)
Phytogenics	*Greek Origanum vulgare subsp. hirtum*; *Sophora japonica L*	Finishing pigs	ADG ↑, G:F ↓, live body weight loss ↓, hot carcass weight ↑, dressing percentage ↑, Opto-star value ↑, pH value ↑, drip loss ↓, reactive oxygen species ↓ and TBARS ↓, and GSH-Px T-SOD ↑ (serum, muscle, and liver)	(Zou et al. 2016b)
Phytogenics	*Origanum vulgare L*. and *Castanea sativa Mill*	Finishing pigs	Hot carcass weight ↑, lipid oxidation ↓, Lightness ↓, redness ↑, H° values↓, cooked meat (taste, color, overall liking), activities in the Longissimus lumborum muscle (TBARS ↓ and GSH-Px, glutathione reductase ↑)	(Ranucci et al. 2014)
Plant extract	Grape seed proanthocyanidin	Finishing pigs	serum, muscle and liver (↑ glutathione, GSH-Px, T-AOC and ↓ MDA), protein levels of Nrf2 ↑, Lipid metabolism (↑ high-density lipoprotein cholesterol levels and ↓serum triglyceride and total cholesterol levels),	(Feng et al. 2023)

## Conclusion

This review focused on phytogenic feed additives as potential substitutes for antibiotic growth promoters in swine production. Natural and phytogenic products have great benefits over antibiotic agents, as well as are profitable and have less chance of resistance development. Therefore, they are considered better alternatives to antibiotic growth promoters, and the use and focus of research on phytogenic products is increasing. The addition of essential oil, plant extract, and herbal extract as phytogenic to swine feed is beneficial for growth performance, nutrient digestibility, biochemical profile, gene expression, hypocholesterolemic, immunity, meat quality, fatty acid composition, amino acid content, and especially in mitigating the impact of disease and environmental stressors on gut health. It has been suggested that the addition of phytogenics to swine feed attenuates the stress response by suppressing the NF-κβ and Nrf2 pathways and enhancing the expression of anti-inflammatory cytokines. This review demonstrates that essential oil, plant extract, and herbal extract could be utilized as phytogenics in the swine industry for improved food safety, health, and economic aspects.

## Data Availability

Not applicable.

## Abbreviations

ADFI: Average daily feed intake
ADG: Average daily gain
AGP: Antibiotic growth promoters
BWG: Body weight growth
CAT: Catalase
CP: Crude protein
DM: Dry matter
E.coli: Escherichia coli
EE: Ether extracts
ERK: Extracellular signal-regulated kinases
G:F: Gain to feed ratio
GE: Gross energy
GIT: Gastrointestinal tract
GnRH: Gonadotropin-releasing hormone
GSH-Px: Glutathione peroxidase
IgA: Immunoglobulin A
IGF-1: Insulin-like growth factor 1
IgG: Immunoglobulin G
IgM: Immunoglobulin M
IL-1: Interleukin 1
IL-10: Interleukin 10
IL-12: Interleukin 12
IL-1β: Interleukin 1β
IL-2: Interleukin 2
IL-6: Interleukin 6
INF-γ: Interferon-gamma
JNK: C-Jun N-terminal kinases
MAPK: Mitogen-activated protein kinase
MCP-1: Monocyte chemotactic protein-1
MDA: Malondialdehyde
MyHC: Myosin heavy chain
NF-kB: Nuclear factor kappa-light-chain-enhancer of activated B cells
NO: Nitric acid
Nrf2: Nuclear factor erythropoietin-2-related factor
OM: Organic matter
SCFA: Short-chain fatty acids
SIgA: Secretory immunoglobulin A
SOD: Superoxide dismutase
T-AOC: Total antioxidant capacity
TBARS: Thiobarbituric acid reactive substances
TGF-β1: Transforming growth factor-β
TLR: Toll-like receptors
TNF-α: Tumor necrosis factor α
TNF-β: Tumor necrosis factor β
TNF-γ: Tumor necrosis factor γ
ZO-1: Zonula occludens-1
